# Biliary Strictures and Cholangiocarcinoma – Untangling a Diagnostic Conundrum

**DOI:** 10.3389/fonc.2021.699401

**Published:** 2021-09-30

**Authors:** Alexander Ney, Andres Garcia-Sampedro, George Goodchild, Pilar Acedo, Giuseppe Fusai, Stephen P. Pereira

**Affiliations:** ^1^ Institute for Liver and Digestive Health, University College London, London, United Kingdom; ^2^ St. Bartholomew’s hospital, Barts Health NHS Trust, London, United Kingdom; ^3^ Division of Surgery and Interventional Science – University College London, London, United Kingdom

**Keywords:** cholangiocarcinoma, primary sclerosing cholangitis (PSC), biliary strictures, ERCP (Endoscopic Retrograde Cholangiopancreatography), early detection, biomarkers

## Abstract

Cholangiocarcinoma is an uncommon and highly aggressive biliary tract malignancy with few manifestations until late disease stages. Diagnosis is currently achieved through a combination of clinical, biochemical, radiological and histological techniques. A number of reported cancer biomarkers have the potential to be incorporated into diagnostic pathways, but all lack sufficient sensitivity and specificity limiting their possible use in screening and early diagnosis. The limitations of standard serum markers such as CA19-9, CA125 and CEA have driven researchers to identify multiple novel biomarkers, yet their clinical translation has been slow with a general requirement for further validation in larger patient cohorts. We review recent advances in the diagnostic pathway for suspected CCA as well as emerging diagnostic biomarkers for early detection, with a particular focus on non-invasive approaches.

## 1. Introduction

Cholangiocarcinomas (CCAs) are a heterogenous and highly aggressive group of tumours which arise from the biliary epithelium (1, 2). Depending on their anatomical location within the biliary tree, CCAs are classified as intrahepatic (iCCA), perihilar (pCCA) or distal (dCCA). Striking differences in their biology and clinical management, challenge the historical classification of pCCA and dCCA under the umbrella term ‘extrahepatic CCA’ (eCCA). Aside from their anatomical localisation, a common feature of CCAs is their poor prognoses, with overall five-year survival rates below 20% (3–5).

CCA is the second most common primary hepatic malignancy, accounting for 15% of all primary liver tumours (6). Epidemiological data suggest a rise in incidence in western countries (0.3–6 per 100,000 people per year) (7), while in some regions of East Asia higher rates (>6 per 100,000) are observed which are associated with liver fluke infections. Albeit a rare disease, CCA is relatively common in those with primary sclerosing cholangitis (PSC); a chronic, fibro-inflammatory and cholestatic liver disease, characterised by progressive fibrosis and biliary stricturing (8, 9). Up to 15% of patients with PSC will develop CCA, with the highest incidence 2-5 years into diagnosis (10). Other common risks include genetic, environmental (liver fluke), lifestyle (alcohol consumption and smoking), chronic infections (HBV and HCV), metabolic syndromes (diabetes mellitus, NAFLD) and obesity, as well as certain chronic inflammatory states (inflammatory bowel disease, chronic pancreatitis) (11). The lack of aetiological factors linked to patients at risk for CCA, however, makes early detection more challenging (12).

Surgical resection or liver transplantation remain the only curative option, with added benefit when followed by adjuvant chemotherapy such as capecitabine (median OS 36.4 vs 51.1 months respectively) (13). Most cases (>70%) are unfortunately non-resectable at time of diagnosis (14, 15), where therapeutic options are limited to systemic therapy and palliation (12, 13, 16). For these cases, first-line gemcitabine plus cisplatin and second-line FOLFOX (folinic acid, 5-fluorouracil and oxaliplatin) is recommended (13). Reports of improved R0 resection rates (>83%) following neoadjuvant chemo-radiotherpay, are limited to small cohort studies and case series, and determination of the exact role of neoadjuvant treatment in the setting of CCA, requires further validation using large sampled - randomized controlled trials (15). CCA tumours often consist of small nests of epithelial cancer cells surrounded by an abundant desmoplastic stroma and a complex tumour microenvironment, formed by cancer-associated fibroblasts, immune cells, endothelial cells and extracellular matrix, making pathological diagnosis challenging (7).

Current diagnostic modalities include clinical, biochemical, radiological and histological techniques – all of which are beset by relatively low sensitivity or specificity, often making the accurate diagnosis of CCA, particularly in patients with PSC, difficult (8, 17). Additionally, none of the currently available tissue or liquid biomarkers are sufficiently sensitive or specific to reliably aid in the early diagnosis of CCA (18). The relatively low incidence of CCA coupled with the high frequency of concomitant cholestasis and cholangitis, as well as difficulties obtaining adequate tissue samples, have hampered the identification of more accurate biomarkers (19).

## 2. Diagnosis and Management of Benign and Malignant Biliary Strictures

Differentiating benign from malignant biliary strictures (BBS and MBS respectively) remains a challenge despite advances in imaging and tissue cytogenetic profiling techniques (20–22). BBS are most commonly iatrogenic in aetiology, and are frequently observed following liver and pancreato-biliary surgery, including cholecystectomy (23–25). The formation of biliary anastomosis is often complicated by strictures which can be a single, localised (anastomotic strictures) or multiple and rather more proximal to the anastomosis site in their non-anastomotic counterparts (23). Other aetiologies of BBS include Inflammatory (i.e. chronic pancreatitis), autoimmune (i.e. PSC and IgG4 sclerosing cholangiopathy), infectious (tuberculosis, parasitic), vascular pathologies as well as radiotherapy induced biliary duct sclerosis (26, 27). MBS arise as consequence of a malignant process within the biliary tree (i.e. CCA), primary or metastatic liver, pancreatic (ductal adenocarcinoma) or ampullary, as well as gallbladder primary neoplasms (25, 28).

**Figure 1:**
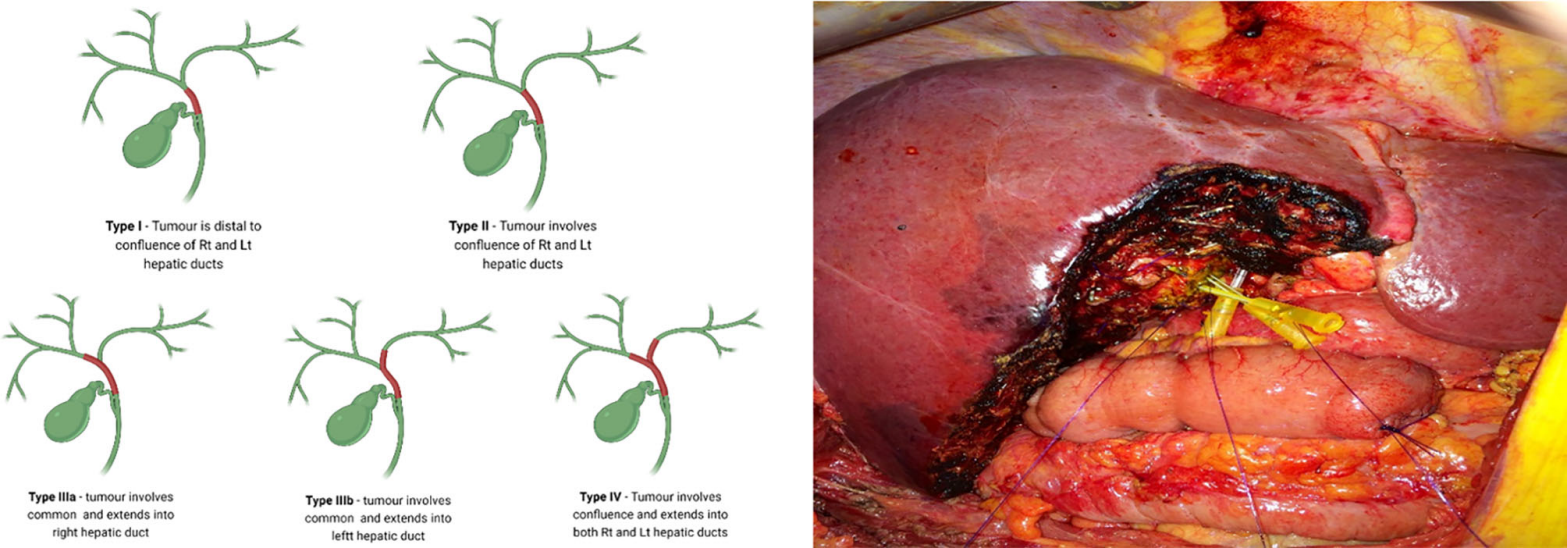
Surgical resection of perihilar CCA is guided by the Bismuth-Corlette classification with types II-IV (Left) requiring extensive surgery, which involves major hepatic resection in the form of extended hepatectomy and caudate lobectomy (Right). (Right) - central hepatic and biliary confluence resection for a Bismuth-Corlette type II hilar CCA. Right anterior, right posterior and Left hepatic ducts are cannulated.

Indeterminate strictures in which laboratory parameters, imaging and histology are inconclusive, would prompt for urgent surgical intervention. The differentiation of malignant from benign peri-hilar strictures in particular, is more challenging (29). Despite the use of a multi-modal approach, and considering the low diagnostic accuracy of existing modalities - the presence of malignancy cannot be excluded on the basis of a negative biopsy with confidence (29). Surgical resection of pCCA is guided by the Bismuth-Corlette classification (30) with types II-IV requiring extensive surgery which involves major hepatic resection in the form of extended hepatectomy and caudate lobectomy (**Figure 1**) (29, 31). Reports of up to 25% of patients who underwent surgical resection for suspected CCA and subsequently found to have benign disease, highlight an urgent need for more accurate diagnostics (32–34). A diagnostic modality with improved sensitivity over standard cytology and histology, may therefore prevent unnecessary extensive surgery which has been linked with morbidity and mortality rates as high as 60% and 18%, respectively (29, 35).

### 2.1 Endoscopic Retrograde Cholangiopancreatography

A number of diagnostic approaches can be applied in the workup of CCA (**Table 1**). Endoscopic retrograde cholangiopancreatography (ERCP) fluoroscopy with brush cytology (ERCP-BC) (and/or forceps biopsy) is the primary sampling technique. However, the predictive value (sensitivity ranging 6-64%, specificity 98-100%) of ERCP-BC is limited by the often inadequate amount of sampled tissue, cellular atypia (due to underlying inflammation or long term biliary stenting), or the well differentiated appearance of certain carcinomas – factors that challenge conclusive differentiation of benign from malignant epithelium (36). The diagnostic utility of ERCP and fine needle aspiration (FNA) is similarly limited by poor sensitivity for the detection of CCA (pooled sensitivity of 45%-65%) and is challenged by location, size and type of lesion, interpretation of cytology and operator skills (20, 37–39). Intraductal biopsy under fluoroscopic guidance may also increase the positive predictive value for CCA during ERCP (45), although this has not been proven in prospective studies for patients with PSC.

**Table 1:** Commonly used imaging modalities for guidance of tissue biopsies in MBS and CCA.

Modality	Differentiates	SEN (%)	SPE (%)	Study
Endoscopic retrograde cholangiopancreatography (ERCP)				
ERCP- Brush cytology (BC)	Benign from malignant (n=539)	6-64%	98-100%	(36)
ERCP-BC	Benign from malignant			
	ERCP-BC vs ERCP-FNA	45%	66%	(37)
	(n=294)	75%	79%	
ERCP – Fine needle aspiration (FNA)	Benign from malignant	45-65%	–	(20, 37–39)
Single operator per-oral cholangioscopy (SOC)	Benign from Malignant (n=539)	71.9%	99.1%	(40)
Endoscopic Ultrasound (EUS)				
EUS – FNA	Benign from malignant	80%	97%	(41)
EUS – FNA	Benign from malignant	75%	79%	(37)
Fluorescence *in situ* hybridisation (FISH) 9p21 polysomy	CCA vs healthy (n=828)	51%	93%	(42)
FISH polysomy 9p21	Benign from malignant (n=281)			(43)
+ cytology		63%	–	
vs cytology alone		35%	–	
	Benign from malignant (n=614)			(44)
ERCP-BC		38.5%	98%	
ERCP-BC and FISH		84.2%	54.1%	
With added cholangioscopic biopsy		80.4%	–	

SEN, sensitivity; SPE, specificity; CCA, cholangiocarcinoma; ERCP, Endoscopic retrograde cholangiopancreatography; BC- Brush cytology; FNA, Fine needle aspiration; SOC, Single operator cholangioscopy; EUS, Endoscopic ultrasound; FISH, Fluorescence in situ hybridisation.

### 2.2 Single Operator Cholangioscopy

**Figure 2:**
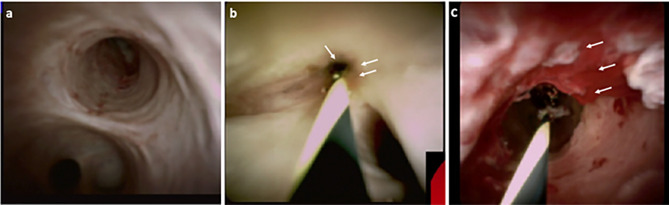
Direct visualisation of the biliary epithelium using cholangioscopy, in healthy **(A)**, PSC stricture **(B)** and CCA **(C)**. Characteristics such as surface irregularity, filling defects, nodularity and neovascularisation (**C** – white arrows) are often indicative of malignancy, with high positive predictive value.

**Figure 3:**
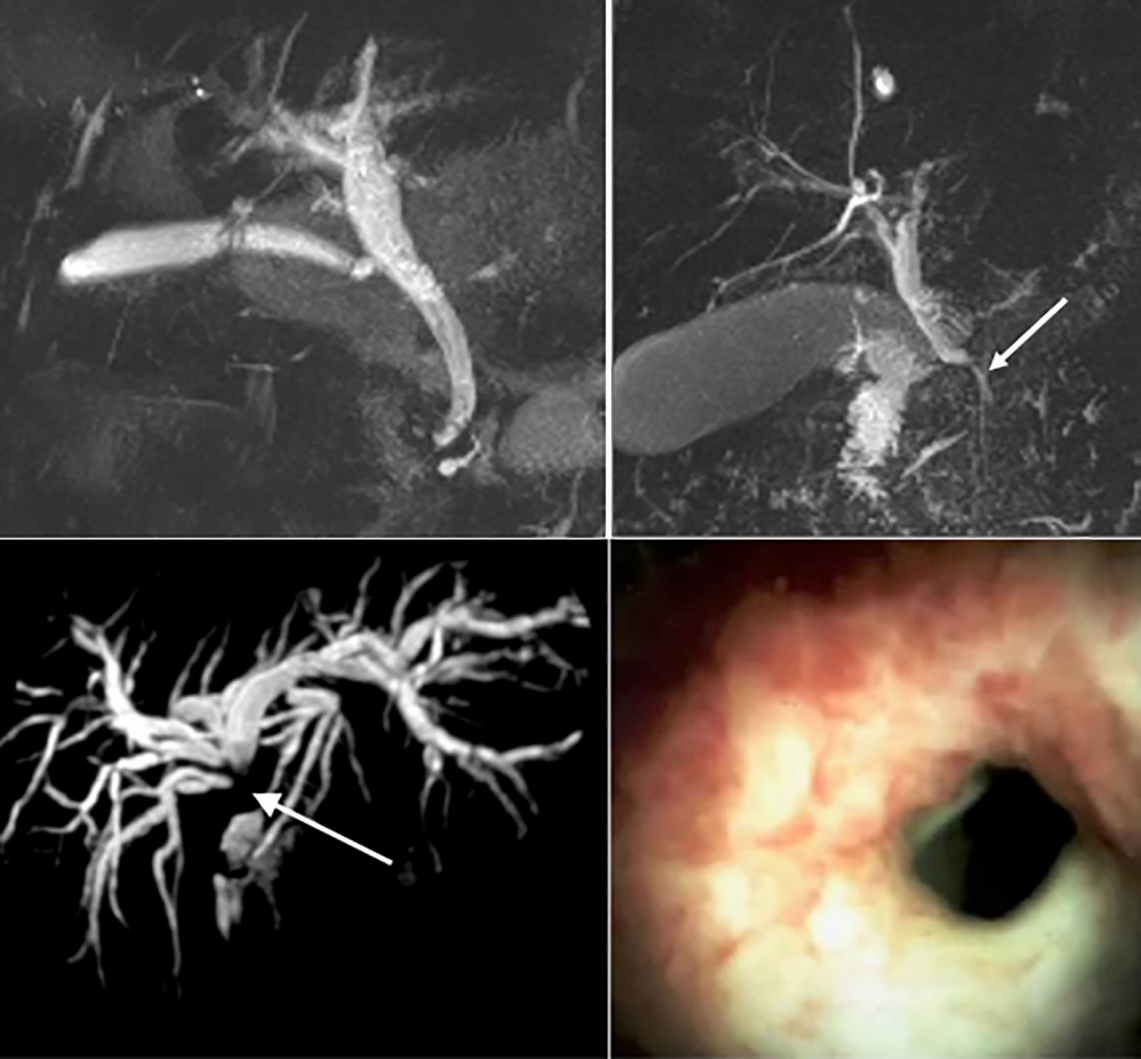
Top left: Magnetic resonance imaging (MRI) of a normal biliary tree. Top right: MRI imaging of a benign biliary stricture secondary to acute pancreatitis (white arrow). Bottom Left: MRI of a dilated intrahepatic biliary tree secondary to a cholangiocarcinoma at the liver hilum (white arrow). Bottom right: Cholangioscopy demonstrating mucosal nodularity and neovascularisation in the 7 -o’clock position, secondary to cholangiocarcinoma.

Cholangioscopy enables the direct visualisation of the biliary epithelium, characterisation of filling defects as well as targeted biopsy of suspicious strictures (**Figures 2**, **3**). Morphological differences between BBS and MBS seen using cholangioscopy (i.e. surface irregularity, nodularity, neovascularisation) are often indicative, with dilated and tortuous vessel presence allowing for positive predictive values as high as 100% (46, 47). Moreover, it allows for minimally invasive ablative techniques (endoscopic, photodynamic and radiofrequency ablation) to be applied, and offers a non-surgical alternative in management of patients with type 2 Mirizzi syndrome (46). Single operator peroral cholangioscopy (SOC) with cholangioscopy-guided biopsy was reported to have a pooled sensitivity and specificity of 66% and 98% in a 2015 systematic review (20). The use of novel optical techniques which augment the visualised mucosa during cholangioscopy is increasing, and these include chromoendoscopy, biliary narrow band imaging and probe-based confocal laser endomicroscopy (pCLE) (48–50). In one prospective study of 136 patients with indeterminate biliary strictures, the addition of pCLE to standard ERCP with brush cytology increased sensitivity from 56% to 89%, with an overall diagnostic accuracy of 88% (51). A more recent meta-analysis of 15 studies which included a total of 539 subjects, reported a pooled sensitivity of 71.9% (95% CI 0.66-0.77) and specificity of 99.1% (95% CI 0.97-0.99) of peroral-cholangioscopy (POC) directed biopsies in the differentiation of malignant from benign strictures (40). This meta-analysis however, did not include any randomised-controlled studies but only reports of small, single centre studies. With respect to PSC related biliary strictures, a report from a single-centre retrospective study showed a limited impact of SOC guided biopsies on clinical management of study subjects, with lower accuracy observed compared to brush cytology (sensitivity and specificities of 15%, 65% and 47%, 95% respectively) (52). In this cohort of 80 patients, however, a higher prevalence of PSC (40%) with previous plastic stenting was described. In these patients, the clinical impact of SOC was limited, and using SOC guided tissue sampling changed management in only 17% of patients (52).

### 2.3 Endoscopic Ultrasound

Considering the low diagnostic yield of ERCP- FNA and forceps biopsy, the use of endoscopic ultrasound (EUS) together with fine needle aspiration as well as intraductal ultrasound (IDUS) (sensitivity and a specificity of 98% in one retrospective study) (53), has gained popularity. The role of the latter in diagnosis of biliary strictures has not yet been established due to it being an emerging modality. EUS-FNA can differentiate benign from malignant strictures with high accuracy (pooled sensitivity and specificity of 80% and 97% respectively) (41), and compared to ERCP-BC, demonstrated significantly improved diagnostic yield. In a prospective study evaluating EUS-FNA alongside ERCP-BC under same sedation in 50 patients with intermediate biliary strictures, Moura et al. reported accuracy of 100% *vs.* 54.8% (p=0.019) for EUS-FNA vs ERCP-BC in the diagnosis of extra-ductal lesions, respectively (54). A comparable diagnostic accuracy was observed with both modalities, in the assessment of intra-ductal or smaller lesion (less than 1.5cm in size) (54). In a meta-analysis which included 294 patients subjected to either EUS-FNA or ERCP-BC for tissue diagnosis of MBS, improved sensitivity and specificity were similarly observed when using EUS for guiding FNA (75% vs 45% and 79% vs 66%, respectively) with accuracies of 79% vs 61% respectively (37). In a meta-analysis of 10 studies evaluating the incremental benefit of EUS-FNA in patients with previously non-diagnostic ERCP-BC, Chiang et al. concluded that EUS has value in the diagnosis of extrahepatic biliary strictures in patients with previously indeterminate strictures following ERCP. Taken together, the data showed that a definitive diagnosis of malignancy was confirmed in an additional 14% of cases when a non-diagnostic ERCP-BC was followed by EUS-FNA (55). The tandem use of these two modalities has shown to improve the diagnostic accuracy of ERCP (around 60-70%) by as much as 30% when EUS-FNA followed. With respect to the nature of the strictures, the highest diagnostic accuracy (97% for both sensitivity and specificity) was observed when a non-diagnostic ERCP was followed by a second ERCP-BC in intrinsic lesions, and by EUS-FNA in case of extrinsic lesions (38, 56). An important consideration emerging from similar studies concerns needle tract seeding, where peritoneal metastases following EUS guided biopsies in CCA and pancreatic cancer patients was observed (57, 58). The risk of needle tract seeding following EUS-FNA however remains unclear (ranging ~2-4%) (57, 59, 60), with several studies describing lack of actual impact on disease progression and overall survival, in both CCA and pancreatic cancer (60–62). Nevertheless, inclusion of the puncture site in those proceeding to surgical resection, reduces the risk of dissemination (62). Real time microscopic diagnosis using CLE allows interrogation of the epithelium with high resolution, using intra-ductal fluorescein dye injection. Due to reduced positive predictive value and specificity when used in combination with ERCP, CLE is reserved for strictures still indeterminate following assessment using POC and IDUS – despite a sensitivity of 98% compared to ERCP alone (45%) (63).

### 2.4 Fluorescence *In Situ* Hybridisation

The addition of fluorescence *in situ* hybridisation (FISH) was shown to improve detection rates. By using fluorescent probes that target specific chromosomal aberrations, the diagnostic accuracy of brush cytology is enhanced (64). Aneuploidy of chromosomes 3, 7, and 17 as well as 9p21 polysomy are most commonly assessed, and have reported to improve the overall sensitivity for detecting malignancy by as much as 50% compared to cytology (43, 44, 65). A systematic review and meta-analysis (SRMA) published in 2014 of eight studies involving 828 patients, demonstrated that the pooled sensitivity and specificity of FISH polysomy to detect CCA was 51% and 93%, respectively (42). Similarly, more recent reports in larger cohorts support the diagnostic benefit of FISH for detection of malignant strictures. Brooks et al., reported their findings in a cohort of 281 patients (49% with underlying malignancy) who were evaluated using either EUS-FNA, or cholnagioscopic biopsy with or without FISH assays. In this study, FISH polysomy 9p21 and cytology had a significantly higher sensitivity compared to cytology alone [63 versus 35% (*p* < 0.05)] (43). EUS-FNA (in distal stricture evaluation) and cholangioscopic biopsy (proximal strictures) increased test sensitivity by 60% (from 33 to 93%; *p* < 0.001) and 48–76% (*p* = 0.05) in previously indeterminate strictures on cytology (43). In a single centre retrospective study of 614 patients, Han et al., reported that the addition of FISH to brush cytology resulted in increased sensitivity for detection of malignancy (84.2% (95% CI 68.8–94%) *vs.* 38.5% (95% CI 31.6–45.8%) observed with BC and fluoroscopic biopsy) (44). The added use of cholangioscopy however, reduced the sensitivity to 80.4% (95% CI 67.6–89.8%). Of note, highest specificity (nearing 98%) was reported with BC alone (95% CI 95.8–98.9%), while the use of FISH reduced test specificity to 54.1% (95% CI 42.9–65%) (44). The low sensitivities in these studies highlight the need for better performing markers for the detection of CCA.

## 3. Diagnostic and Prognostic Biomarkers in CCA

### 3.1 Disease Markers in Clinical Use: CA19-9, CEA and CA125

Biomarkers can be applied in the diagnosis, prognostication and management of a given disease. An ideal biomarker allows early disease detection with high sensitivity and specificity, is technically simple to obtain and quantify - at an acceptable cost. No such biomarker for the detection of cholangiocarcinoma, has been identified so far. Moreover, considering the low incidence as well as the heterogenous clinical picture at presentation of biliary tract cancers, screening the general population for CCA is neither feasible nor cost effective. Biomarkers aimed at differentiating high risk biliary tract lesions associated with CCA in clinical use include carbohydrate-antigens 19-9, 125 (CA19-9 and CA125 respectively) and carcino-embryonic antigen (CEA). CA19-9 is the primary tumour marker used in the diagnosis of CCA, although its lack of specificity and limited sensitivity hampers its clinical utility. A meta-analysis included 1,264 patients with CCA reported pooled sensitivity and specificity of 72% and 84%, respectively, for the differentiation of CCA from healthy controls and those with PSC (AUC 0.83) (66). Furthermore, in the 5-10% of the general population who are Lewis (a) antigen negative, CA 19-9 will remain undetectable (67). CA19-9 is elevated in a variety of hepatic and extra-hepatic conditions including cholangitis and cholestasis, further limiting its use in PSC, where these conditions commonly co-exist (7, 68). One retrospective study of 79 patients with PSC found that more than one-third of patients with a dominant biliary stricture and CA 19-9 >129 IU/ml did not have CCA after a median follow-up of 30 months (69). Due to the limited sensitivity of CA19-9 in the detection of CCA, any result must be interpreted in the context of the clinical picture and findings from cross-sectional imaging (70). Current screening strategies for patients with PSC may include annual magnetic resonance cholangiopancreatography (MRCP) (**Figure 3**) coupled with serum CA19-9, although this surveillance strategy is not currently recommended in the European Society of Gastroenterological Endoscopy (ESGE) (70) or British Society of Gastroenterology (71) guidelines, due to a lack of supportive outcome data.

CEA is a cell-surface anchored glycoprotein that is involved in cell adhesion. Its elevation is most often associated with colorectal malignancies, however, it is raised in up to 30% of patients with CCA showing a sensitivity and specificity of 72% and 84%, respectively (12). CA125 is a membrane associated glycoprotein encoded by the MUC16 gene which is often elevated in ovarian cancer, although it is also raised in up to 50% of CCA cases (72, 73).

### 3.2 Bile and Tissue Markers of CCA

#### 3.2.1 Markers in Bile

Nucleic acids, proteins, circulating tumour cells as well as extracellular vesicles can be obtained from various bodily fluids (e.g. serum, bile, urine, saliva) or tissue, with variable diagnostic values in the context of CCA. The presence of tumour specific proteins (which are either shed or directly secreted by the malignant biliary epithelium), as well as tissue specific oncogenic proteins (e.g. enzymes) and their metabolites (onco-metabolites) make bile a particularly valuable reservoir of disease markers. The presence and levels of such diagnostic targets can be assessed using omics-based analyses, in bile obtained at time of endoscopic assessment (**Table 2**).

**Table 2:** Bile and tissue markers.

Biomarker	Differentiates	Fluid	SEN (%)	SPE (%)	AUC	Study
**Bile acids**						
Conjugated **vs.*.* Unconjugated	CCA (n=16) *vs.* BBD (n=29)	Bile	88.9%	87.1%	–	(74)
**Proteins and peptides**						
ADAMTS4 KLK4, KLK6 CASP1 CMA1	CCA (n=52) *vs.* BBD (n=76)	Bile Urine	–	–	0.96	(75)
LTF, NT5E, CPM, A2M, ACTN4	CCA (n=36) *vs.* BBD (n=36)	Bile	100%	100%	1.0	(76)
MCM-5	CCA *vs.* BBD (n=97)	Bile	65.4%	78%	–	(77)
Elastase, amylase	CCA (n=22) *vs.* BBD (n=28)	Bile	82%	89%	-	(78)
**Extracellular vesicles**						
miRNA191, U486-3p, U1274b, U16, U484	CCA (n=46) *vs.* PSC (n=13) *vs.* BBD (n=37)	Bile	67%	97%	-	(79)
**miR-191, miR-486-3p, miR-1274b and miR-484**	CCA (n=5) *vs.* PDAC (n=20) *vs.* BBD (n=10)	Bile	100%	100%	1.0	(80)
**Tissue miRNA** miRNA-21	CCA (n=18) *vs.* healthy (n=18), BBD	Tissue	95%	100%	0.995	(81)

SEN, sensitivity; SPE, specificity; AUC, area under (ROC) curve; CCA, cholangiocarcinoma; PSC, primary sclerosing cholangitis; BBD, benign biliary disorders.

##### 3.2.1.1 Metabolites and Proteins

Specific changes in the composition of bile can both induce and be suggestive of an underlying malignant process in the biliary tree (11, 82, 83). A reduction in total biliary bile acid content was noted by several groups as a unique feature in CCA, compared to other malignant (PDAC) and benign causes of biliary obstruction (76, 83–85). Differences between conjugated to unconjugated-bile acid ratios, can be used in the differentiation of these pathologies with a relatively high accuracy (88.9% sensitivity and 87.1% specificity) (74). Specifically, Song et al., suggested a role for glycocholic acid (GCA) and taurochenodeoxycholic acid (TDCA) as specific to CCA with a higher average composition ratio of GCA (35.6%), compared to patients with pancreatic cancer or other benign biliary conditions (85). The higher concentrations of GCA and TDCA in states of chronic cholestasis, regardless of their aetiology, stimulate malignant cell proliferation, invasion and downregulation of apoptosis, through receptor mediated activation of the (MAPK) ERK 1/2 and PI3k/AKT signalling cascades, among others (11, 86). Secondly, the activation of receptors mediating these signalling pathways [i.e. G-protein coupled bile acid receptor 1 (GPBAR1) and sphingosine 1 phosphate receptor 2 (S1PR2), or nuclear factor kappa B (NF-κB)] in CCA tissue samples is enhanced (82, 86–89). Furthermore, differences between serum/stool ratios of GCA and tauroursodeoxycholic acid (TUDCA), have been linked with a specific composition of intestinal microbiota (*Lactobacillus*, *Actinomyces*, *Peptostreptococcaceae*, and *Alloscardovia*) as a feature in iCCA *vs.* patients with liver cirrhosis, HCC, as well as compared to healthy subjects (90).

Proteomic profiling of bile can distinguish CCA from PSC and non-malignant conditions such as biliary tract lithiasis with high accuracy (91–93). Peptide fingerprints of three proteases identified in bile - ‘a disintegrin and metalloproteinase with thrombospondin motifs 4′ (ADAMTS4), kallikrein-4 (KLK4) and chymase (CMA1) differentiate CCA from benign strictures (*p* <  0.05). When combined with a urinary panel (ADAMTS4, proteases caspase-1 (CASP1) and KLK6), these proteins differentiated CCA patients (n=36) from PSC (33) and others with benign conditions (n=18), as observed in a case-control phase II study on 87 patients (AUC 0.96 at 95% CI 0.89 to 0.99) (75). Using an artificial intelligence- machine learning based approach to biomarker discovery, Urman et al., reported diagnostic utility of two 5-protein based biomarker panels identified in bile (and urine) taken from patients with benign (*n* = 36) and malignant conditions, CCA (*n*=36) or PDAC (*n*=57). The biliary panel (LTF, NT5E, CPM, A2M, and ACTN4) differentiated patients with benign cholangiopathy and CCA with an AUC of 1 (at 100% sensitivity and 100% specificity). The urinary panel included MUC5B, FAT4, ALB, AMY2A and ENPP7 which allowed differentiation of PDAC patients from controls with similarly high accuracy (AUC 1, at 100% sensitivity and 100% specificity) (76).

##### 3.2.1.2 Mini-chromosome Maintenance Proteins

Mini-chromosome maintenance (MCM) proteins have key roles in DNA replication, and are known markers of oncogenesis and cancer progression (94–97). Ayaru et al., reported the value of biliary MCM-5 as a marker of pancreato-biliary malignancy with a sensitivity of 66% compared to 20% for brush cytology (97). Similar sensitivity (65.4%) of biliary MCM-5 in differentiating malignant (n=50) from benign strictures (n=47), compared with 25% for brush cytology, was reported by Keane et al. (77). Levels of certain pancreatic enzymes (elastase, amylase) have also been reported as altered in patients with CCA (compared to benign obstructive aetiologies) and showed a high sensitivity (82%) at 89% specificity in their differentiation (78).

##### 3.2.1.3 Extracellular Vesicles and Micro RNAs

Extracellular vesicles (EVs) are cell secreted exosomes, microvesicles, or apoptotic bodies which contain biomolecules such as nucleic acids, proteins, as well as lipids. EVs encase their cargo within a lipid membrane which keeps them stable in various bodily fluids, from which they can be isolated for diagnostic purposes (98, 99). EVs act as means of inter-cellular communication and in cancers, mediate tumour formation and shaping of metastatic niche landscapes (100). EVs have been found to retain key membrane markers from their cell of origin, making them excellent candidates for cancer detection (101). One of the first studies reporting the value of bile derived nucleic acids as a diagnostic tool in biliary pathologies included a panel of five miRNAs (191 *U* 486‐3p *U* 1274b *U* 16 *U* 484). In this study, which was published in 2014, a panel sensitivity of 67% at 96% specificity, highlighted the value of miRNAs in differentiating malignancy (n=46) and PSC (n=16) from benign cholestatic conditions (n=34) such as BBSs, papillary stenoses, choledocholithiasis, pancreatic cysts and cholangitis (79).

Severino et al., showed that the total amounts of EVs isolated from bile samples obtained from CCA patients exceed those identified in BBSs - a finding which can be explained by the high metabolic turnover in malignancy (80). Furthermore, the authors described five outstanding miRNAs (miR-191, miR-486-3p, miR-1274b and miR-484) which were identified in bile samples taken from patients with CCA, pancreatic cancer (5 and 20 patients respectively), and from those with chronic pancreatitis (n=15) and biliary stones (n=10), at time of ERCP. An exceptional accuracy in differentiating CCA from other benign strictures (100% sensitivity and 100% specificity) was reported (80).

#### 3.2.2 Tissue Markers

##### 3.2.2.1 Genomic Alterations in CCA

The mutational landscape of CCA is heterogeneous although alterations in some genes have a higher prevalence. The differential abundance of metabolites or proteins can guide toward a diagnosis of CCA. Striking changes in the metabolic profile of glucose, lipids and nucleotides can already be observed in early disease stages (84). These can be explained by the high metabolic demand involved in constitutive activation of proliferative (e.g., RAS, MAPK, PI3/AKT/mTOR) and inflammatory (e.g. STAT3) pathways, as well as loss of tumour suppression mechanisms (e.g. TP53, CDKN2A, BAP1) as part of tumorigenesis (5, 84, 102). Fibroblast growth factor receptor (FGFR) fusions have been described in 10-15% of iCCA (103), but not in other subtypes, and lead to a constitutive activation of the receptor and its signalling pathway, deregulating key processes such as cell proliferation, survival and migration (103, 104). Targeted inhibition (using pemigatinib) in previously treated iCCA patients with FGFR2 gene alteration (fusions/rearrangements), showed objective response in 35.5% of patients compared to patients with other FGFR alterations or FGFR negative controls. The promising results of this phase 2 study (FIGHT-202), are suggestive of a role for FGFR inhibition in selected cases where iCCA is driven by FGFR2 aberrations (104). The benefit of combining pemigatinib with gemcitabine and cisplatin as first-line therapy in unresectable or metastatic CCA is under assessment in a multi-centre phase 3, randomized controlled trial (FIGHT-302; NCT03656536). Enhanced activation of inflammatory signalling pathways (cytokine, chemokine and dendritic pathways) as well as an interplay with certain cytokines (e.g. IL-4, IL-10, IL-17) and their transducer STAT3 – is characteristic of an inflammatory biological subclass of CCA. The enrichment of EGF (BRAF mutations), RAS (KRAS mutations), AKT, VGF and MET proliferative pathways (p<0.05) and specific DNA aberrations (11q 13.2 and 14q22.1) on the other hand, are features in the genetic signature of a proliferative subtype (p<0.001) (102). A survival and disease recurrence analysis in tissue samples from 149 patients with CCA (38% inflammatory and 62% proliferative subtypes), revealed unfavourable outcomes in those patients with a proliferative molecular class (24.3 vs 47.2 months in the inflammatory class; *P* = .048) (102).

##### 3.2.2.2 Metabolites and Proteins

Tissue specific markers (metabolites, enzymes) of glucose, lipid or nucleotide metabolism (e.g. GLUT2, HK2, GFAT, PKM2 and LDHA) enable differentiation between hepatobiliary neoplasms and healthy tissue. Mutations in certain genes that code for metabolic enzymes (i.e. IDH1; isocitrate dehydrogenase 1 - α-ketoglutarate metabolism) or differences in metabolite levels (e.g. glucose, glucose-6-phosphate) allow more accurate differentiation between primary biliary tract and hepatic cancers (84). Mutations in IDH1 and IDH2 are present in 10-20% of CCA patients (105), and result in the accumulation of 2-hydroxyglutarate (2-HG)-increasing oxidative stress (106). Specific genetic signatures can also differentiate between intra and extra- hepatic CCA. Higher frequency of alterations in FGFR, IDH1, IDH2, BAP1, PBRM1, MCL1, CDKN2A, BRAF and BRCA1/2 are observed in iCCA, while KRAS, TP53, CDKN2B, SMAD4, ErbB2 (HER2), CTNNB1 and MLH1 mutations are more suggestive of eCCA (107, 108). Similarly, the presence of specific mutation can be used for the purpose of staging and disease prognostication. Transcriptomic studies in large patient cohorts (137 in one and 292 in a second) assessing the prognostic utilities of KRAS, TP53 and IDH mutations, demonstrated worse prognosis and higher iCCA recurrence in the presence of the former two compared to IDH mutations (109, 110).

##### 3.2.2.3 Mucin Glycoproteins

The abundance of cancer associated proteins in biopsies are other predictors of disease outcomes. The differential expression of several mucin glycoproteins (MUC1, MUC2, MUC4, MUC5) can be used for prognostication in CCA (111–113). A meta-analysis which included 4,126 CCA patients highlighted MUC1 and MUC4 as reliable predictors of survival (HR 2.52; 95 % CI 1.49–4.26, and HR 2.45; 95 % CI 1.56–3.86, respectively), especially when combined with EGFR (HR 1.79; 95 % CI 1.14–2.8), fascin (HR 2.58; 95 % CI 1.19–5.58) and the cell cycle marker p27 (HR 0.29; 95 % CI 0.14–0.6) (114).

##### 3.2.2.4 The Role of Cadherins in CCA

A downregulation of cadherins is observed as part of the epithelial to mesenchymal transition (EMT) process, promoting CCA progression through the TGF-*β axis* (115, 116). Cadherin-17 (CDH17) plays a key role in the development of the gastrointestinal and pancreato-biliary systems (117). Aberrant expression of CDH17 is a feature in various gastrointestinal malignancies including stomach, colon, pancreatic and liver, and is a marker of advanced disease and poor outcomes (117–119). A recently reported multi-variate analysis in a cohort of 180 CCA patients, identified CDH17 elevated expression as a predictor of malignancy as well as a reliable predictor of postoperative survival. Interestingly, a positive correlation with nodal (p=0.04) and distant disease spread was noted, which outperformed widely used disease staging systems (p=0.04) (TNM, LCSGJ and Okabayashi) (117).

##### 3.2.2.5 mIRNA Tissue Profiles Predict Malignancy and Metastatic Disease

Tissue microarrays-based RNA profiling revealed diagnostic as well as prognostic utilities for miRNAs in CCA. miRNAs are non-coding nucleotide sequences (RNA) which are key regulators in carcinogenesis (120). miRNA -191, miRNA-29a and miRNA 21/221 have been implicated in haematological as well as gastrointestinal (pancreatic, gastric, CCA) malignancies, and can be detected in both serum and tissue of patients (120–123). Similarly, their expression levels have been described as independent risk factors in CCA with implications on survival and disease progression (120). miRNA-21 outstands across various reports as a differentiator between biliary tract cancers and healthy tissue (**Table 2**) (81, 121, 123–126). The diagnostic utility of miRNA-21 demonstrated a 95% sensitivity and 100% specificity in distinguishing CCA patients from healthy and benign controls (AUC 0.995) (81). The expression of miRNA-21 can be indicative of metastatic spread (*P* = 0.037) and shorter survival (*P* < 0.05) in liver fluke induced CCA (125).

### 3.3 Novel Blood Biomarkers of CCA

#### 3.3.1 Proteins

Serum pre-fractionation prior to mass-spectrometry enables the detection of low-abundance proteins, which are often masked by highly abundant serum proteins such as globulin and albumin. Analyses of the serum proteome in blood samples from healthy, PSC and CCA have shown changes in serum protein composition which were diagnostic, albeit only in small cohorts of patients (2) (**Table 3**).

**Table 3:** Blood (serum, plasma) biomarkers.

Biomarker	Differentiates	Fluid	SEN (%)	SPE (%)	AUC	Reference
***Proteins/Cytokines***						
OPN	CCA (n=107) *vs.* Healthy (n=55)	Serum	88.0	100.0	0.964	(127)
Fucosylated fetuin A	CCA (n=39) *vs.* PSC (n=39)	Serum	62.0	90%	0.815	(128)
IL-6	CCA (n=26) *vs.* Healthy (n=23)	Serum	73.0	92.0	0.875	(129)
CA50	iCCA (n=85) *vs.* [Healthy + BBD + OC] (n=116)	Serum	65.9%	87.3%	0.806	(130)
RvD1	CCA (n=31) *vs.* BBD (n=27)	–	–	–	0.783	(131)
MMP-7	CCA (n=44) *vs.* BBD (n=36)	Serum	75.0	78.0	0.730	(132)
CYFRA 21-1 MMP-7 CEA CA19-9	CCA (n=24) *vs.* BBD (n=25)	Serum	92.0	96.0	–	(133)
CYFRA 21-1 PKM2 MUC5AC GGT	CCA (n=66) *vs.* PSC (n=62)	Serum	81.8	90.0	0.903	(134)
AFP	iCCA (n=45) *vs.* HCC (n=76)	Serum	91.1	55.3	–	(135)
AFP CA-242	iCCA (n=45) *vs.* HCC (n=76)	Serum	93.4	89.7	–	(135)
AFP GPC3	iCCA (n=36) *vs.* HCC (n=210)	Serum	88.10	82.68	0.836	(136)
CA19-9 CA-S27 CCA-CA WFA+-MUC1 WFA+-M2BP	CCA (n=138) *vs.* [Healthy + OV + BBD + OC] (n=246)	Serum	80.4	81.7	0.873	(137)
S100A6	CCA (n=29) *vs.* Healthy (n=22)	Serum	86.0	91.0	0.909	(138)
DKK1	CCA (n=37) *vs.* Healthy (n=50)	Serum	76.0	100.0	0.872	(139)
SSP411	CCA (n=35) *vs.* BBD (n=13)	Serum	90.0	83.0	0.913	(140)
***Cell-free non-coding RNA***						
miR-21	iCCA (n=25) *vs.* Healthy (n=7)	Serum	–	–	0.910	(141)
miR-21	iCCA (n=25) *vs.* Healthy (n=7)	Plasma	–	–	0.940	(141)
miR-21	BTC (n=94) *vs.* Healthy (n=50)	Plasma	85.1	100.0	0.93	(142)
miR-21	BTC (n=94) *vs.* BBD (n=23)	Plasma	72.3	91.3	0.83	(142)
miR-21	BTC (n=75) Benign (n=20) Healthy (n=68)	Serum	–	–	0.7 0.724
miR-221	BTC (n=75) Benign (n=20) Healthy (n=68)	Serum	–	–		(143)
miR-150	CCA (n=35) *vs.* Healthy (n=35)	Serum	91.43	80.0	–	(144)
miR-150 CA19-9	CCA (n=35) *vs.* Healthy (n=35)	Serum	93.33	96.88	–	(144)
miR-483-5p miR-222	CCA (n=40) *vs.* PSC-derived CCA (n=40)	Serum	–	–	0.770	(145)
miR-1281	CCA (n=31) *vs.* PSC (n=40)	Serum	55.0	90.0	0.830	(146)
miR-126	CCA (n=31) *vs.* PSC (n=40)	Serum	68.0	93.0	0.870	(146)
miR-26a	CCA (n=31) *vs.* PSC (n=40)	Serum	52.0	93.0	0.780	(146)
miR-30b	CCA (n=31) *vs.* PSC (n=40)	Serum	52.0	88.0	0.780	(146)
miR-122	CCA (n=31) *vs.* PSC (n=40)	Serum	32.0	90.0	0.650	(146)
***Metabolites***						
TSA	CCA (n=89) *vs.* [BBD+Healthy] (n=81)	Serum	71.9	81.4	0.856	(147)
TSA	CCA (n=69) *vs.* HCC (n=59)	Serum	82.6	83.1	0.885	(148)
21-deoxycortisol Bilirubin LysoPC (14:0) LysoPC (15:0)	CCA (n=225) *vs.* Healthy (n=101)	Serum	98.5	99.2	0.993	(149)
***Protein-containing EVs***						
AMPN	CCA (n=43) *vs.* Healthy (n=32)	Serum	90.7	65.6	0.878	(150)
VNN1	CCA (n=43) *vs.* Healthy (n=32)	Serum	72.1	87.5	0.876	(150)
PIGR	CCA (n=43) *vs.* Healthy (n=32)	Serum	83.7	71.8	0.844	(150)
FIBG	CCA (n=43) *vs.* PSC (n=30)	Serum	88.4	63.3	0.796	(150)
A1AG1	CCA (n=43) *vs.* PSC (n=30)	Serum	76.7	70.0	0.794	(150)
S100A8	CCA (n=43) *vs.* PSC (n=30)	Serum	69.8	66.6	0.759	(150)
FCN2	Early stage CCA (I-II) (n=13) *vs.* PSC (n=30)	Serum	100.0	80.9	0.956	(150)
ITIH4	Early stage CCA (I-II) (n=13) *vs.* PSC (n=30)	Serum	91.7	80.9	0.881	(150)
FIBG	Early stage CCA (I-II) (n=13) *vs.* PSC (n=30)	Serum	91.7	80.9	0.881	(150)
***RNA-containing EVs***						
RFFL	CCA (n=12) *vs.* [PSC+UC+healthy] (n=23)	Serum	100.0	100.0	1.00	(151)
ZNF266	CCA (n=12) *vs.* [PSC+UC+healthy] (n=23)	Serum	91.7	91.3	0.976	(151)
OR4F3	CCA (n=12) *vs.* [PSC+UC+healthy] (n=23)	Serum	100.0	87.1	0.960	(151)
miR-551B	CCA (n=12) *vs.* [PSC+UC+healthy] (n=23)	Serum	83.3	87.0	0.909	(151)
PMS2L4	CCA (n=12) *vs.* [PSC+UC+healthy] (n=23)	Serum	91.7	87.0	0.880	(151)
LOC643955	CCA (n=12) *vs.* [PSC+UC+healthy] (n=23)	Serum	83.3	87.0	0.873	(151)
***EV surface markers***						
AnnexinV**^+^** EpCAM**^+^** AS6PR1**^+^**	CCA (n=26) *vs.* Liver disorders (n=53)	Serum	65.8	47.0	0.621	(152)

SEN, sensitivity; SPE, specificity; AUC, area under (ROC) curve; CCA, cholangiocarcinoma; PSC, primary sclerosing cholangitis; BBD, benign biliary disorders; iCCA, intrahepatic cholangiocarcinoma; HCC, hepatocellular cholangiocarcinoma; OC, other cancers; OV, infection of Opisthorchis viverrini; BTC, biliary tract cancer (includes CCA, bile duct cancer, gallbladder cancer and ampulla of vater cancer); EV, extracellular vesicles; UC, ulcerative colitis.

Osteopontin is a matricellular protein previously linked with multiple types of cancer and implicated in several pathological processes such as inflammation and tumorigeneses (153). Loosen et al., reported elevated levels of osteopontin in sera of patients with CCA (n=27) compared to samples taken from patients with PSC (n=10) (H test p = 0.001) (127). Remarkably, elevated levels of circulating osteopontin were associated with poor survival following tumour resection.

Investigators from the Mayo Clinic performed serum glycomic and proteomic analysis on 117 patient samples, 39 of which had CCA and 39 - PSC. In this study Betesh et al., identified differences in expression of certain glycans in blood taken from patients with CCA. One protein, fucosylated fetuin A, was able to differentiate CCA patients from those with PSC with reasonable performance (AUC 0.815 versus AUC 0.63 for CA19-9), suggesting a role for this protein in the surveillance of patients with PSC (128).

Anti-glycoprotein (GP)-2 immunoglobulin A autoantibodies are associated with CCA development in patients with PSC (154). In a European cohort of 250 patients, anti-GP2 positivity in PSC patients was associated with a significantly higher risk of developing CCA, independently of disease duration, bilirubin level and age (154).

Different cytokines identified in sera of CCA patients have shown diagnostic and/or prognostic values. The pro-inflammatory cytokine interleukin 6 (IL-6) was found elevated in serum samples of patients with CCA compared to healthy individuals, with test sensitivity and specificity of 73% and 92%, respectively (129). IL-6 however, can also be elevated in other hepatobiliary cancers like HCC, accentuating the need for more CCA specific cytokines (12).

Carbohydrate antigen 50 (CA50) is a cancer-associated cell surface antigen known to be expressed in malignancies of the digestive tract, including pancreatic and colorectal cancers, but has also been reported to be elevated in cirrhosis, pancreatitis or type 2 diabetes mellitus (155). In a recent study, serum levels of CA50 taken from 85 patients with iCCA were compared to healthy individuals (n=110), patients with benign biliary disorders (n = 23) and other cancers (n = 33). CA50 levels differentiated iCCA from non-CCA cases with 65.9% sensitivity and 87.3% specificity (AUC = 0.806) (130).

Another study linked lower levels of the circulating anti-inflammatory agent resolving D1 (RvD1) with CCA. Patients with CCA had lower levels (<380 ng/mL) of RvD1 when compared to serum levels in patients with benign biliary disorders (AUC = 0.783) – with suggested correlation with disease stage (131). In this study however, RvD1 did not perform any better than CA19-9 in the same cohort of patients (AUC = 0.940).

Elevated levels of matrix metalloproteinase 7 (MMP-7), an enzyme with key roles in extracellular matrix remodelling during tissue repair and tumour progression, have been found in the serum of patients with CCA. A study by Leelawat and co-workers compared the diagnostic potential of MMP-7, with CEA and CA19-9 in differentiating CCA patients from those with benign biliary tract diseases. MMP-7 showed better diagnostic value (AUC 0.730) compared to CEA and CA19-9 (AUC of 0.63) in this report (132).

Combinations of proteins into multi-marker panels have been reported to increase their individual diagnostic performance by several authors. A study by Lumachi et al. compared the individual performance of serum cytokeratin-19 fragment (CYFR21.1), MMP-7, CEA and CA19-9 and in combination, in the detection of CCA. Serum levels of these proteins in twenty-four patients with histologically confirmed CCA and 25 matched patients with benign liver were measured. The mean value of each marker was significantly higher (p<0.01) in CCA compared to controls and the combination of all serum markers was reported to have test sensitivity of 92% at 96% specificity for detecting CCA, with an overall diagnostic accuracy of 94% (133).

More recently, our group evaluated a number of biomarkers with diagnostic utility in the differentiation of CCA from benign biliary disease. In a cohort of 66 patients with CCA and 62 with PSC, a panel combining serum levels of PKM2, CYFR21.1, MUC5AC and GGT was able to differentiate CCA from PSC with test sensitivity of 82% and specificity of 90% (AUC 0.90) (134).

Another proposed CCA biomarker is serum alpha fetoprotein (AFP). In one study, AFP levels differentiated HCC from iCCA with 91.1% sensitivity but only at 55.3% specificity. Combination of AFP with carbohydrate antigen 242 (CA-242) increased test specificity to 93.4%, albeit with lowered sensitivity (135). More recently, another study tested the combination of AFP with serum glypican-3 (GPC3) to differentiate HCC from iCCA in a larger cohort of patients (n=210 and n=36 respectively). The results showed that even though test sensitivity was increased compared to AFP alone (67.62% to 88.10%), the overall accuracy did not improve (AUC 0.836 to 0.853) (136).

The performance of a novel biomarker panel combining five cancer-associated glycans and glycoproteins, (known as glycobiomarkers or “GlycoBiomarker (GB)-score”), has been recently evaluated in patients with CCA. More specifically – levels of CA19-9, carbohydrate antigen-S27 (CA-S27), CCA-associated carbohydrate antigen (CCA-CA), WFA-positive MUC1 (WFA+-MUC1), and WFA-positive M2BP (WFA+-M2BP) were measured in serum taken from 138 CCA patients and 246 non-CCA controls, showing a 80.4% sensitivity and 81.7% specificity (AUC = 0.873) (137).

Other serum proteins have been reported as stage specific and prognostic in CCA. A recent study analysed the serum proteome of 148 HCC and 60 CCA patients by liquid chromatography-tandem mass spectrometry (LC-MS/MS) and found 25 differently expressed proteins in CCA patients, with AUC ranging from 0.701 to 0.823 (156). Interestingly, the authors reported that levels of plasma serine protease inhibitor inversely correlated with tumour development (stage I to IV). In addition, levels of afamain - a human plasma vitamin E-binding glycoprotein, and previously described ovarian cancer tumour marker (157), were associated with poor prognosis in CCA (156). Despite their potential, these similarly require further validation in larger and randomized cohorts.

#### 3.3.2 Serum Metabolites

Specific changes in the concentration of serum metabolites can also point towards a diagnosis of CCA. Quantitative metabolomic analyses of serum taken from patients with CCA identified a role for serum metabolites as biomarkers of this cancer. Total sialic acid (TSA), a nine-carbon sugar present in oligosaccharide chains of many glycoproteins and glycolipids, has been associated with CCA development. In a study comparing serum TSA of CCA patients and a control group formed by healthy individuals and patients with benign biliary conditions, the sensitivity and specificity were 71.9% and 81.4% respectively (AUC 0.856) (147). Similarly, Kongtawelert et al. compared TSA levels in CCA and HCC patients and reported an overall AUC of 0.885 (148), highlighting the relevance of this metabolite in CCA detection.

Lastly, serum metabolomes of a larger group of CCA patients were compared with those of healthy (n = 176 and 85, respectively), and identified 75 differently expressed metabolites between these cohorts. As reported by Liang et al., following further validation (n=225), 21-deoxycortisol, lysophosphatidylcholine 14:0 (lysoPC 14:0), lysophosphatidylcholine (lysoPC 15:0) and bilirubin (over-expressed) (the latter 2 over-expressed in CCA), were selected based on their superior diagnostic performance (AUC 0.918, 0.954, 0.927 and 0.922). The combination of these four markers into a panel showed an even higher diagnostic accuracy (AUC of 0.993) (149).

#### 3.3.3 Liquid Biopsies

The presence of specific genetic signatures which are identified using targeted genomic analysis of tumour tissue, are increasingly used for diagnosis, prognostication and evaluation of treatment response (158, 159). Image guided tissue acquisition for diagnosis confirmation is required pre-operatively, yet is frequently limited by insufficient tissue yield which is required for accurate molecular profiling. Specifically, in the presence of a highly fibrotic tumour stroma, the retrieval of adequate tissue material which sufficiently captures the heterogeneity of biliary cancers, is imperative. Technological advancements in genomic analysis have enabled the identification of genetic (and epigenetic) aberrations in blood, through isolation of tumour-derived circulating nucleic acids (circulating free DNA (cfDNA), cell free RNA) or tumour cells (CTCs) (159). Known as ‘liquid biopsy’, such tests are increasing in popularity, as they provide a minimally invasive access to a rich source of cancer derived genetic material which can be used for diagnosis and monitoring of tumour evolution and progression (159).

##### 3.3.3.1 Cell-Free Non-Coding RNA

Micro-RNAs (miRNAs) are small, highly conserved RNA molecules involved in post-transcriptional regulation of genes (160). An important characteristic of miRNAs is their presence and stability in biofluids, which can often be sampled less invasively. Researchers have reported key roles for miRNAs in the pathogenesis (proliferation, invasion and metastasis) of various cancers, including CCA (161). In one small study, miR-21 and miR-221 were found to be significantly overexpressed in plasma (AUC 0.94) and serum (AUC 0.91) of patients with iCCA (n=25), compared to healthy controls (n=7) (141). Similarly, miR-21 differential expression in tissue, showed outstanding diagnostic performance (AUC=0.995) (81) in differentiating CCA from benign disease and healthy controls (141). A similar study confirmed the value of serum miR-21 in differentiating CCA from healthy controls (AUC 0.93), as well as CCA from benign biliary disease including PSC (AUC 0.83) (142). However, miR-21 was also found upregulated in other cancers including HCC, questioning its specificity (162). Outstanding from a panel of five miRs (miR-10a, miR-21, miR-135b, miR-221, and miR-214), exosome derived miR-221 levels alone differentiated malignant (n=75) from benign (n=20) and 68 healthy samples (p<0.01) in a recent study published by Han et al. (143). The authors suggested a role for miR-221, in the early detection of BTCs (143).

Serum level differences of miR-150 demonstrated test sensitivity and specificities of 91.43% and 80%, respectively, when measured in CCA and compared to healthy controls, with improved performance when assayed together with CA19-9 (93.33% sensitivity and 96.88% specificity) (144). A similar observation was reported by Wang et al. (163). A small study which included 30 patients with PSC and 30 patients with CCA, found that serum miR-483-5p and miR-222 show different expression patterns in PSC compared to CCA cohorts (145). Another study by Voigtländer and collaborators, reported five other miRs (miR-1281, -126, -26a, -30b, -122) to be differentially expressed in serum of patients with PSC and CCA (ROC AUCs ranging from 0.70 to 0.91), although their combination did not significantly improve diagnostic accuracy (146).

Whilst most reported diagnostic miRs appear to be up-regulated in CCA, others such as miR-150-5p or miR-106a may be down-regulated (164, 165).

##### 3.3.3.2 Circulating Tumour Cells

Circulating tumour cells (CTCs) are released into the blood stream by primary tumours, and their serum levels showed both diagnostic and prognostic value in several malignancies including HCC, gastric, pancreatic, breast as well as colorectal cancers (166–170). However, their low abundance limit their use; even in metastatic settings, CTCs represent as little as one out of 109 of total circulating cells (171). Despite these, investigators further evaluated the diagnostic potential of CTCs in the context of CCA. A number of technologies have been developed in an attempt to isolate CTCs from peripheral blood, including the CellSearch^®^ System, which is licenced by the US Food and Drug Administration. This semi-automated platform identifies, isolates and enumerates CTCs using cell specific EpCAM antibodies and immunofluorescent markers. The often lack of sufficient expression of EpCAM by tumours is however a disadvantage. In one study, up to 20% of CCAs did not overexpress this protein (172). CellSearch^®^ further differentiates cell types based on their positivity to DAPI, cytokeratins (such as 8, 18 and 19) or negativity to CD45 (173). One early study demonstrated the ability of this system to detect CTCs in patients with metastatic cancer versus healthy controls and those with benign disease. Of 344 patients that were either healthy or had an underlying benign disease, only 0.3% had >2 CTCs per 7.5ml of blood, compared to 36% of specimens collected from patients with metastatic disease, although it is not stated whether any had metastatic CCA (174). In another small study of 13 patients with CCA reported by Al-Ustwani et al., only 3 patients had significantly elevated number of CTCs (>2 per 7.5ml of blood) in this small cohort (173). Other studies have suggested that levels of CTCs may be associated with a poor prognosis in patients with advanced CCA (175, 176), however, their diagnostic role in CCA is yet to be fully determined.

#### 3.3.6 Extracellular Vesicles

In a study performed by Arbelaiz et al., serum EVs from healthy controls and patients with CCA (n=43), PSC (n=30) and hepatocellular carcinoma (HCC), were isolated and characterised. These were supplemented by EVs previously derived from human CCA cell lines and normal cholangiocytes *in vitro* (150). Proteomic analysis revealed that the proteins with best performance in differentiation of CCA from healthy controls were AMPN, VNN1 and PIGR, showing AUCs of 0.878, 0.876 and 0.844 respectively. Additionally, several differentially expressed proteins were identified in serum EVs of CCA versus PSC patients, including FIBG, A1AG1 and S100A8 (maximum AUC 0.80). Some candidates (including FCN2, ITIH4 and FIBG) also showed higher diagnostic values for early stage CCA (stages I-II) versus PSC than CA19-9 (AUC 0.956, 0.881 and 0.881, respectively), showing the potential usefulness of these serum EV proteomic signatures, as early diagnostic tools in CCA. Further validation in larger cohorts are however pending.

Lapitz and colleagues performed transcriptomic analysis of the content of serum EVs isolated from CCA patients, and a control group which included PSC, ulcerative colitis and healthy individuals (151). EV contained miRNAs RFFL (E3 ubiquitin-protein ligase rififylin), ZNF266 (zinc finger protein 266) and OR4F3 (olfactory receptor family 4 subfamily F member 3) showed the best diagnostic performance (AUC 1.00, 0.976 and 0.960, respectively). With respect to non-coding RNAs, miR-551B, PMS2L4 and LOC643955 distinguished these patients with highest accuracies (AUC 0.909, 0.880 and 0.873, respectively).

Lastly, Julich-Haertel et al. used a different approach which was based on the identification of membrane bound markers of large EVs or tumour-associated microparticles (taMPs). The group compared the level of serum taMPs containing specific surface markers in patients with liver malignancies (including CCA and HCC) and non-liver cancers and cirrhosis. taMPs carrying Annexin V, EpCAM and ASGPR1 showed a sensitivity of 65.8% but low specificity of 47.0% (AUC 0.621) (152).

### 3.4 Urinary Biomarkers of CCA

#### 3.4.1 Volatile Organic Compounds

Urine is an excellent sample matrix as it is non-invasively accessible, can be obtained in large quantities and is stable in its composition if handled correctly. Urine is source abundant in volatile organic compounds (VOCs) which can differentiate CCA from benign biliary conditions. Selected-ion flow-tube mass spectrometry (SIFT-MS) allows the measurement of their concentration in urine. Navaneethan et al., showed that ethane and 1-octene distinguished CCA from PSC patients with an AUC of 0.90 (80% sensitivity at 100% specificity) (177). Other VOCs such as 2-propanol and acetonitrile have also been reported as having value in differentiation CCA from patients with PSC or other benign lesions (AUC 0.862) (177).

#### 3.4.2 Proteins and peptides

Furthermore, since urine is an ultrafiltrate of plasma, the urinary proteome is highly sensitive to changes in renal function and a wide range of non-renal diseases, including certain cancers (178). Urinary proteomic biomarkers have been described in many tumours including pancreatic, renal, prostate, bladder, lung, breast, ovarian cancer and CCA (179). Metzger and colleagues used capillary electrophoresis-mass spectrometry to evaluate the urinary proteome in early CCA (180). A 42-biomarker panel was initially identified based on the differentially excreted urinary peptides of 41 patients including 14 with CCA, 13 with PSC and 14 with other benign biliary disease. In a subsequent cross-sectional validation of 123 patients, the urinary peptide panel accurately diagnosed 35 of 42 CCA patients and 64 of 81 patients with benign biliary disease (including those with PSC), with an AUC of 0.87, 83% sensitivity and 79% specificity. Evaluation of 101 healthy controls gave 86% specificity. More recently, combined bile and urine proteome analysis was performed in a case-control phase II study of 87 patients (36 CCA, including 13 with CCA on a background of PSC, 33 PSC and 18 other benign disorders). A logistic regression model was developed and subsequently validated in a prospective cohort of 45 patients undergoing ERCP for the evaluation of biliary strictures (181). The combination of both urine and bile markers gave an accuracy of 92% in the detection of CCA (sensitivity 94%, specificity 76%, AUC 0.84). Other groups have suggested that urinary miRNAs may be useful in the diagnosis of CCA. In a recent study of 192 patients with either *Opisthorchis viverrini* infection, periductal fibrosis or CCA, miR-21 and miR-192 were found to be elevated in the urine of patients with CCA versus healthy controls (AUC 0.849). Of these two biomarkers, miR-21 discriminated CCA with most accuracy (AUC 0.682) (182). **Table 4** summarises the performance of urinary2 biomarker panels in the diagnosis of CCA.

**Table 4:** Urinary biomarkers.

Biomarker	Differentiates	Fluid	SEN (%)	SPE (%)	AUC	Reference
**Volatile** **Compounds**						
2-propanol, Acetonitrile	CCA (n=6) *vs.* PSC (n=10) *vs.* BBD (n=29)	Urine	–	–	0.862	(177)
Ethane, 1-Octane	CCA (n=6) *vs.* PSC (n=10)	Urine	80%	100%	0.90	(177)
**Urinary peptides, proteins**						
42- peptide panel	CCA (n=52) *vs.* PSC/BBD (n=80)	Urine	83%	79%	0.87	(181)
Combined urine/bile peptide panel	CCA (n=52) *vs.* PSC/BBD (n=80)	Urine	94%	76%	0.84	(181)
MUC5, FAT4, ALB, AMY2A, ENPP7	PDAC *vs.* healthy	Urine	100%	100%	1.0	(76)
**Extracellular** **vesicles**						
miRNA-21	CCA (n=22) *vs.* healthy (n=21)	Urine	63.6%	71.4%	0.68	(182)
miRNA-192	CCA (n=22) *vs.* healthy (n=21)	Urine	63.6%	66.7%	0.68	(182)
**mRNAs** INO80D, RRAGD MAP6D1	CCA (n=23) *vs.* healthy (n=5)	Urine	–	–	1.0	(151)
**Non-coding**	CCA (n=23) *vs.* healthy (n=5)	Urine				(151)
**RNAs**			–	–	0.90	
MIR200C, HCG4			–	–	0.93	
LOC100134868			–	–	0.89	
**mRNAs** CLIP3, VCAM1 and TRIM33	CCA *vs.* PSC	Urine	–	–	0.96	(151)
**mRNAs**	CCA (n=23) *vs.* PSC, UC, Healthy (n=22)	Urine	–	–		(151)
MT1F			–	–	0.915	
GPX3			–	–	0.897	
LDHA				–	0.894	

SEN, sensitivity; SPE, specificity; AUC, area under (ROC) curve; CCA, cholangiocarcinoma; PDAC, Pancreatic ductal adenocarcinoma; PSC, primary sclerosing cholangitis; BBD, benign biliary disorders; UC, Ulcerative colitis.

Recent interest in EV isolation from body fluids (such as serum and bile) has also included urine as a diagnostic target. Urine isolated EVs can be screened using transcriptomics showing distinct signatures of CCA as compared to patients with PSC and healthy controls. Lapitz et al., identified specific messenger RNAs [INO80D, RRAGD and MAP6D1 (AUC 1.00)] as well as certain non-coding RNAs [MIR200C, HCG4 and LOC100134868; AUC of 0.904, 0.930 and 0.896, respectively)] in EVs isolated from urine of patients with CCA (n=23), differentiating them from healthy controls (n=5). The authors have also reported higher expression of CLIP3, VCAM1 and TRIM33 messenger RNAs in CCA versus PSC (AUC 0.965), while others such as MT1F, GPX3 and LDHA were able to distinguish CCA from a mixed cohort of patients with PSC, ulcerative colitis and healthy patients with high accuracy (AUC 0.915, 0.897, 0.894, respectively) (151).

## 4 Concluding Remarks

CCAs are a group of heterogenous malignancies which are a devastating form of cancer that is most often diagnosed at a late stage. Due to their aggressiveness, the only curative option for all subtypes is radical surgical resection, which is often supplemented with adjuvant chemotherapy (13). CCA is however most often diagnosed at a non-resectable stage, where associated 5-year survival is less than 5%. The exact role of neoadjuvant chemotherapy in improving oncological resection rates has not yet been fully established, and larger scale trials are required for validation of the few studies that have showed benefit (15). The early detection of CCA remains a major challenge, particularly in patients with PSC and currently available diagnostic modalities are not sufficiently sensitive to differentiate malignant from benign strictures with ideal accuracy (20, 37–39, 183). Tissue, cytological and bile-based biomarkers may provide additional diagnostic information over standard methods, with variable sensitivities and specificities - ranging from 58%-87% and up to 98%-100%, respectively (183). Owing to technological developments in the field of genomics, molecular profiling of lesions and identification of tumour specific genetic and epigenetic alterations, can guide toward diagnosis (and appropriate differentials), prognosis, as well as direct treatment (159). Similarly, the evolvement of proteomic and metabolomic techniques and their application in biliary tract cancer research, identified striking changes in protein or metabolite compositions in tissue and bile, which can be used to differentiate malignant from benign biliary lesions. However, these ‘invasive’ markers can often only be obtained by subjecting patients to invasive procedures, such as ERCP with biliary brush cytology or EUS with biopsy, therefore their use in screening is limited. In attempts to minimise the invasiveness of such diagnostic tests, molecular signatures and metabolic changes in other bodily fluids (such as blood an urine) have also been investigated. Although showing only modest diagnostic accuracies at best, these findings which have been reported in small cohorts, justify further interrogation in the form of larger scale validation studies.

Liquid biopsies in which tumour derived genetic material can be isolated from blood (circulating DNA, RNA or circulating tumour cells), offer an appealing and minimally invasive approach for both diagnostic (and surveillance) purposes, as well as means for monitoring treatment response. Various nucleic acids (e.g.miRNAs), freely circulating or exosome bound, and CTCs with potential theranostic utilities have been described, although many still require validation in collaborative studies (5). Among these, mIR-21 and miR-221 levels have been commonly reported to have diagnostic value across several studies (141–143, 182). The correlation of such novel biomarkers with CA19-9 levels is likely to further improve their diagnostic accuracies (81, 143). Advances in molecular profiling and sampling techniques have improved researchers’ understanding of CCA evolution. Moreover, recent evidence points to improved clinical outcomes, when therapeutic targeting of specific genetic aberrations (e.g. FGFR fusions) in selected cohorts of patients, is guided by tumour genomic analyses (104, 108). Despite increasing reports of biomarker studies in CCA, these have mostly been observed in limited numbers of subjects and clinical samples. Their translation into clinic however, requires larger cohort validations - which are challenged by the low incidence of this devastating subtype of cancer.

## Author Contributions

AN, concept, literature search, writing and edits, revision of manuscript. AG-S, literature search, writing of manuscript. GG, literature search, writing of manuscript. PA, critical revision and edits, supervision. GF, critical revision and supervision. SP, concept, supervision and critical revision of manuscript. All authors contributed to the article and approved the submitted version.

## Conflict of Interest

The authors declare that the research was conducted in the absence of any commercial or financial relationships that could be construed as a potential conflict of interest.

## Publisher’s Note

All claims expressed in this article are solely those of the authors and do not necessarily represent those of their affiliated organizations, or those of the publisher, the editors and the reviewers. Any product that may be evaluated in this article, or claim that may be made by its manufacturer, is not guaranteed or endorsed by the publisher.
